# Cotton Rats Alter Foraging in Response to an Invasive Ant

**DOI:** 10.1371/journal.pone.0163220

**Published:** 2016-09-21

**Authors:** Andrea K. Darracq, L. Mike Conner, Joel S. Brown, Robert A. McCleery

**Affiliations:** 1 Department of Biology, University of North Georgia, 151 Sunset Blvd., Dahlonega, GA, United States of America; 2 Joseph W. Jones Ecological Research Center, 3988 Jones Center Dr., Newton, GA, United States of America; 3 Department of Biological Sciences, University of Illinois – Chicago, 845 West Taylor Street, Chicago, IL, United States of America; 4 Department of Wildlife Ecology and Conservation, University of Florida, 110 Newins-Ziegler Hall, P.O. Box 110430, Gainesville, FL, United States of America; University of Missouri Kansas City, UNITED STATES

## Abstract

We assessed the effects of red imported fire ants (*Solenopsis invicta*; hereafter fire ant) on the foraging of hispid cotton rats (*Sigmodon hispidus*). We used a manipulative experiment, placing resource patches with a known amount of millet seed within areas with reduced (RIFA [–]) or ambient (RIFA [+]) numbers of fire ants. We measured giving up densities (the amount of food left within each patch) within the resource patches for 4 days to quantify the effects of fire ants on cotton rat foraging. We assessed the effects of fire ant treatment (*RIFA*), *Day*, and their interaction on cotton rat giving up densities. Giving up densities on RIFA [+] grids were nearly 2.2 times greater across all foraging days and ranged from 1.6 to 2.3 times greater from day 1 to day 4 than the RIFA [–] grids. From day 1 to day 4, mean giving up densities decreased significantly faster for the RIFA [–] than RIFA [+] treatments, 58% and 13%, respectively. Our results demonstrate that cotton rats perceive a risk of injury from fire ants, which is likely caused by interference competition, rather than direct predation. Envenomation from ants likely decrease the foraging efficiency of cotton rats resulting in more time spent foraging. Increased time spent foraging is likely stressful in terms of the opportunity for direct injury and encounters with other predators. These indirect effects may reduce an individual cotton rat’s fitness and translate into lowered population abundances.

## Introduction

An organism’s perception of risk may lead to behavioral (e.g. movement, foraging, habitat selection) and physiological (e.g. stress) changes [1–5] that have population level consequences. In fact, these indirect effects of predators can have a greater influence on prey populations than direct mortality [6]. Prey species have been shown to strongly alter their foraging behaviors based on their perceptions of risk [4,7,8]. To balance the energy gained from foraging with potential risks, foragers may change their temporal use of food resources, increase search time within less risky food patches, and alter their use of microhabitats [5,9]. Alterations in foraging behaviors may in turn influence populations and communities by affecting predator-prey dynamics, competitive interactions, food web interactions, and prey fitness [4,10].

There is an extensive body of literature examining predation risk and foraging in terrestrial systems; but research has mainly focused on native vertebrate predators and their effects on smaller prey. Yet, insects are the most diverse group of non-microbial organisms [11] and they may provide unusual, yet very real, risks of predation or injury (e.g. envenomation) to vertebrates. Specifically, ants (Formicidae) comprise 15–25% of the animal biomass in most terrestrial ecosystems [12]. Several ant genera are proficient predators that employ strategies of biting, and venom injection via a stinger. Additionally, ants use chemical deterrents when threatened or defending territories [13]. Within this context, invasive ants are becoming an increasing problem worldwide, with five species listed among the top 100 worst invasive species [14]. All five species consume seeds as a part of their diet and have displaced native ants [15–19]. Few studies address the influence of invasive ants on native vertebrates that consume seeds (e.g. [20,21]), although it is likely that biting and stinging ants, such as the red imported fire ant (*Solenopsis invicta*; hereafter fire ant), present a risk of injury and/or predation to granivorous rodents.

Fire ants were introduced into the southeastern United States in the 1930s and have been implicated in the decline of several native species [22,23]. Through interference or exploitative competition [24], fire ants may influence ground-foraging rodents, which can be important seed dispersers and prey within ecosystems [25]. Quantifying the effects of fire ants on rodent foraging has implications for plant communities and other ecosystem level processes (e.g. nutrient cycling, predator-prey interactions; [25]). In a laboratory setting, one study demonstrated that deer mice (*Peromyscus maniculatus*) foraged food patches less in the presence of fire ants [21]. Additionally, a separate study found a negative correlation between the presence of fire ants and foraging of oldfield mice (*Peromyscus polionotus)*, which was dependent on microhabitat conditions and precipitation [20].

We assessed the effects of red imported fire ants (*Solenopsis invicta*; hereafter fire ant) on the foraging of hispid cotton rats (*Sigmodon hispidus*) by experimentally excluding fire ants from large field plots. In the presence of fire ants cotton rats altered their maternal care behaviors [26]. Additionally, in the absence of other predators, fire ants decrease the survival of cotton rats [27]. Subsequently, we predicted that the presence of fire ants poses a foraging cost to cotton rats and would cause them to have higher giving-up densities in experimental food patches.

## Materials and Methods

### Study site and study species

Our study was completed within a mature stand of longleaf pine—wiregrass (*Pinus palustris—Aristida stricta*) savannah located on Ichauway, the 12,000 ha research site of the Joseph W. Jones Ecological Research Center. The stand is managed with biennial prescribed fires. Our experiment commenced approximately one year post-fire. Mark-recapture live-trapping from 2012 to 2014 showed that cotton rats and cotton mice (*Peromyscus gossypinus*) were the dominant small mammal species. During the period of our foraging experiments cotton rats were the most abundant species comprising 83% of captures over 3,456 trap nights (S1 Table). The home range size of cotton rats is 0.22 ha and 0.39 ha for females and males, respectively, and their diet consists primarily of grasses [28].

### Experimental design

For our food patches and fire ant treatments, we used six 12 x 12 trapping grids with 10 m spacing that were established in and had been sampled for small mammals seasonally since July 2012. As controls, we randomly selected three grids to be maintained at ambient fire ant numbers (RIFA [+]; n = 39 food patches). We used the remaining three grids for fire ant reductions (RIFA [–]; n = 39 food patches). Specifically, we hand broadcasted 1.7 kg/ha of a granular insecticide (Amdro^**®**^) onto the treatment grids and an approximately 100 m buffer around each grid (10 ha total) to reduce fire ant numbers [29]. Our experimental protocols followed recommendations of the American Society of Mammalogists’ Guidelines for the use of wild mammals in research [30] and were approved by the University of Florida’s Institutional Animal Care and Use Committee (IACUC Approval number 201408514).

**Fig 1:**
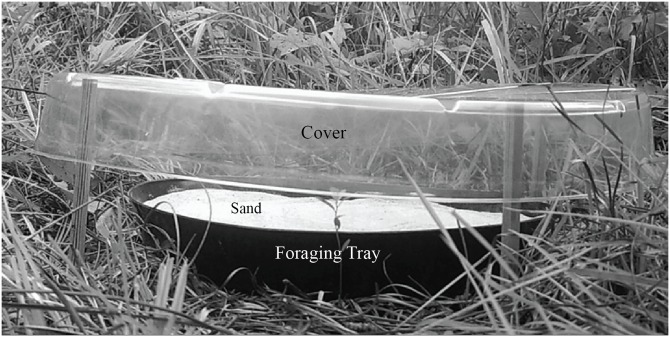
The resource patch design used to assess the influence of red-imported fire ants (*Solenopsis invicta*) on hispid cotton rats (*Sigmodon hispidus*) over four days from 13–15 August 2015.

From 12–13 August 2014, we arrayed 13 resource patches within each grid. We drew food patch stations randomly from the trapping stations with the constraint that food patch stations be at least 30 m apart. We located the resource patches in understory vegetation, primarily wiregrass, approximately 1 m from shrub (woody vegetation > 0.5 m tall) cover. Each resource patch consisted of a 35.6 cm black plastic plant saucer placed on the ground, hereafter referred to as the foraging tray (Fig 1). We elevated a 43.2 cm clear plastic saucer (hereafter cover) on four wooden dowels above the foraging tray, approximately 15.2 cm above the ground (Fig 1). To keep the cover stable above the foraging tray, we inserted two metal stakes on either side of the cover and bent a 15.2 cm portion of the stake at a 90-degree angle over the top of the cover. The cover protected the foraging tray from rain and discouraged foraging from non-target species (e.g. birds). Our foraging substrate consisted of 1 L of sand, collected on our study site, sifted, and poured into each foraging tray.

### Resource patches

We prebaited the resource patches with millet seed for three nights from 13–15 August 2015. Though cotton rats are herbivores and primarily consume grasses, we were interested in their behavioral response to resource patches, which should apply regardless of diet. Following prebaiting, we removed the remaining millet seed and thoroughly mixed 2 g of millet seed (Arrowhead Mills, Boulder, CO) into the sand substrate of each tray. The millet seed had been dried at 60**°**C for 6 h prior to use. For four consecutive nights (15–20 August 2015) we collected (using a sieve) and replaced the millet seed within each resource patch. We also noted signs of foraging such as feces and/or tracks, and whether feces were likely from a mouse or a rat. Rat feces within the tray were likely from cotton rats as they were the only rat species ever caught from June 2012 through December 2014 (38,016 trap nights; A. Long, Unpublished data). In the laboratory, we dried the samples at 60**°**C for 6 h, removed all debris, and weighed each sample to obtain the giving up density (GUD; the amount of food remaining within the patch) at each resource patch each night [31].

### Mark-recapture

Within the same season as the foraging experiment (summer), we sampled rodent populations on each grid (Georgia Department of Natural Resources Scientific Collecting Permit # 29-WJH-13-164). Each mark-recapture session consisted of four nights of trapping. We sampled grids as pairs (a control and a treatment) on 22–25 July, 22–25 August, and 13–16 September 2014. During each session, we placed a Sherman trap (H.B. Sherman Traps, Inc., Tallahassee, FL) baited with oats and birdseed at each grid intersection (144 traps/grid). We checked the traps each morning, closed them, and then reopened them in the evening to avoid any small mammal mortalities related to temperature. Each trapped individual was identified to species, ear tagged (National Band and Tag Company, Newport, KY) in both ears, sexed, weighed, and assessed for reproductive condition (IACUC Approval number 201408456).

### Ant sampling

To quantify the efficacy of the RIFA treatment at resource patches, we used a handmade aspirator to collect the ants within each foraging tray (N = 39 samples per treatment) on the last day of GUD sampling (20 August 2015). We identified and counted all RIFAs within each sample.

### Statistical analyses

We measured GUDs within the resource patches to quantify the effects of fire ants on cotton rat foraging [31]. Under this framework, foragers were expected to use a patch until the harvest rate no longer exceeds the energetic, predation and/or risk of injury, and missed opportunity costs of foraging [31]. Consequently, GUDs are expected to be greater within resource patches where foragers perceive more risk, harassment or hazards [31]. We calculated the GUD as the final weight of millet seed remaining in each resource patch [31]. For trays that had not been foraged, we set the GUD to 2.0 g, the initial weight of millet seed placed into each tray. If we were unsure if a foraging tray had been used (e.g. no feces but possible tracks), we considered GUDs of <1.9 g to indicate that a tray had been foraged. If the GUD was > 1.9 g, we considered the foraging tray unused and set the GUD to 2.0 g.

For each trapping grid, we calculated the minimum number known alive (MNKA) of cotton rats (S1 Table). We limited analyses to cotton rats as they were the most abundant species within the grids and all foraging sign at the patches belonged to cotton rats. The RIFA [–] grids had a greater abundance of cotton rats than RIFA [+] grids. Consequently, we used two approaches to separate density effects on GUDs from the influence of fire ants. If fewer cotton rats utilized more patches in the RIFA [+] grids then rats should have exhibited characteristics of "cream skimmers" [32] and 1) foraged more patches per individual and left higher GUDs, 2) the overall amount harvested per individual should have been greater on RIFA [+] grids, and 3) if rats foraged the same number of patches per individual in the RIFA [+] and RIFA [–] grids, then GUDs from those patches that were foraged should not have differed regardless of fire ant treatment if the GUDs were strictly influenced by density.

To test if fewer rats foraged more extensively in the RIFA [+] grids, we ran a Chi-square test of heterogeneity to assess the null hypothesis that the number of patches foraged was independent from the number of rats available to forage within each treatment (the MNKA). To determine if the overall amount harvested per individual was greater on the RIFA [+] grids, we calculated the amount harvested per individual as [(1.9 g—Mean GUD) * 13) / MNKA] for each grid on each day (S1 Table). We ran an analysis of variance (ANOVA) to test for the interaction effect of *RIFA* and *Day* on the amount of millet seed harvested per rat using *Grid* as the error term in our model. After assessing the potential effects of density, we evaluated the interactive effects of *RIFA* and *Day* on the GUD within a resource patch using an ANOVA (S1 Table). We included the resource patch (*Patch*) nested within *Grid* as an error term in our model.

We assessed effectiveness of the treatment at reducing RIFA numbers using a generalized linear mixed model with a Poisson distribution (S1 Table). We included *RIFA* as the independent variable, the number of RIFA at each station (*Count*) as the dependent variable, and *Grid* as a random variable in our model. We completed all statistical analyses in Program R Version 2.3.1 [33] and considered α = 0.10 to indicate a significant difference.

## Results

**Fig 2:**
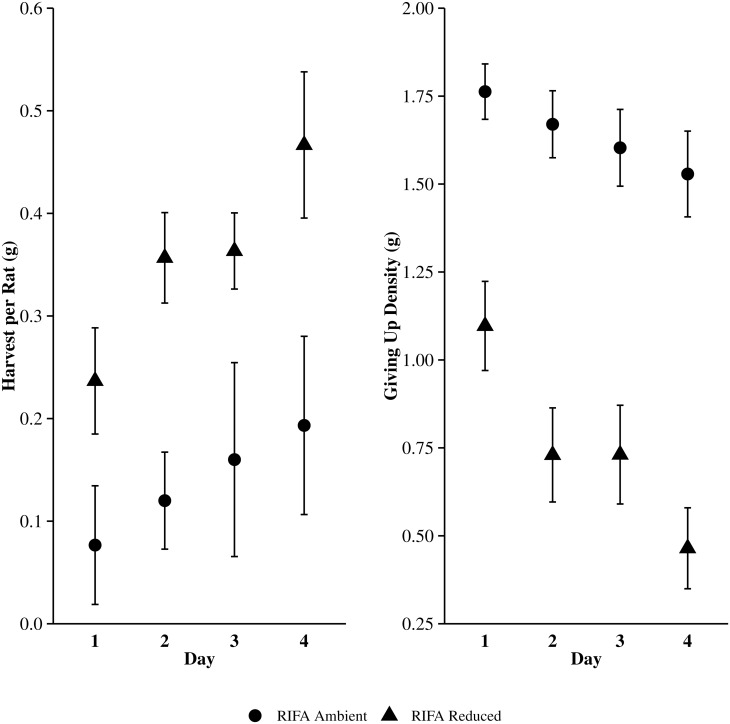
The mean amount harvested per individual (g) and giving up densities (g) of millet seed harvested by hispid cotton rats (*Sigmodon hispidus*) within resource patches located in areas with ambient (RIFA[+]; n = 39) or reduced (RIFA[–]; n = 39) red-imported fire ant (*Solenopsis invicta*) numbers over four days from 13–15 August 2015.

In 3,456 trap nights, we captured 84 (21, 32, and 31 per grid) and 130 (23, 62, 45 per grid) cotton rats on the RIFA [+] and RIFA [–] grids, respectively. Although 17% of our captures were cotton mice (*Peromyscus gossypinus*) and oldfield mice (*Peromyscus polionotus*), cotton rats were the only observed foragers based on sign at the foraging trays. Our analyses to determine if density influenced GUDs indicated that 1) rats did not forage more patches per individual on the RIFA [–] grids compared to RIFA [+] grids (*x*^2^ = 0.19, df = 1, P-value = 0.6646), 2) the overall amount harvested per individual was greater on RIFA [–] compared to RIFA [+] grids (Fig 2, Table 1), and 3) the amount harvested per individual from foraged patches within the RIFA [+] and RIFA [–] grids varied, with the harvest per individual within RIFA [–] grids being nearly 2.6 times greater than the RIFA [+] grids (Fig 2, Table 1).

**Table 1:** The degrees of freedom (DF), mean-squares, F-ratios, and P-values associated with mixed effect analysis of variances (ANOVAs) used to quantify the effects of fire ant treatment (RIFA), day (Day), and their interaction (RIFA * Day) on the amount harvested per individual (g) and giving up densities (g; in log scale) of millet seed harvested by cotton rats (*Sigmodon hispidus*) within resource patches located in areas with ambient (RIFA[+]; n = 39) or reduced (RIFA[–]; n = 39) red-imported fire ant (*Solenopsis invicta*) numbers over four days from 13–15 August 2015.

Source	DF	Mean-square	F-ratio	P-value
Amount harvested per individual				
*RIFA*	1	0.29	7.09	0.0562
*Day*	3	0.03	9.81	0.0015
*RIFA* * *Day*	3	0.00	1.12	0.3806
Error: *Grid*	4	0.04		
Error: Within	12	0.00		
Giving up densities (GUDs)				
*RIFA*	1	131.15	8.26	0.0453
*Day*	3	9.94	25.63	< 0.0001
*RIFA* * *Day*	3	3.24	8.36	< 0.0001
Error: *Grid*	4	15.87		
Error: *Grid* (*Patch*)	72	2.80		
Error: Within	228	0.39		

The variables *RIFA*, *Day*, and their interaction influenced cotton rat GUDs (Fig 2, Table 1). GUDs on RIFA [+] grids were nearly 2.2 times greater across all foraging days and ranged from 1.6 to 3.3 times greater from day 1 to day 4 than the RIFA [–] grids (Fig 2, Table 1). The mean GUD decreased by 13% and 58% from day 1 to day 4 for the RIFA [+] and RIFA [–] treatments, respectively (Fig 2, Table 1).

The number of RIFAs within resource patches on RIFA [+] grids was 23.5 times greater ($\overset{-}{x}\;=\;$ 38.6, S.E. = 6.18) than on RIFA [–] grids ($\overset{-}{x}\;=\;$ 1.64, S.E. = 0.26; β = 4.02, S.E. = 0.95, z = 4.21, p = < 0.001).

## Discussion

At the individual level, fire ants pose a foraging cost (likely due to harassment, bites and stings) that raises GUDs, perhaps increases foraging costs, and reduces the amount of seeds that can be profitably harvested. Fire ants are aggressive when defending their mounds or food resources and may display interference competition towards cotton rats via stinging and subsequent envenomation [22,34]. Other studies have established that envenomation by fire ants may lead to death or have consequences for the long-term survival of vertebrates (e.g. [35–37]) and some species increase their movements in response to fire ants [38, 39, 40]. Likewise, cotton rats may increase their movements within and between food patches to avoid the costs associated with being stung by fire ants. Additionally, fire ants may compete exploitatively with cotton rats for seeds, although we could not address this with our study because ants were unable to remove seeds from food patches (A.K. Long, Personal observation). These individual effects potentially translate into lower population sizes for the cotton rats when faced with fire ants (cotton rats were c. 50% more abundant on the fire ant removal grids).

Cotton rats are a substantial component of the diets of mammalian (e.g. Bobcat [*Lynx rufus*]; [41], avian (e.g. Northern harrier [*Circus cyaneus*]; [42], and reptilian (e.g. Eastern coachwhip [*Masticophis flagellum*]; [43]) predators throughout their range. Prey should reduce mobility in response to perceived predation risk [5,44], but envenomation from ants likely decrease the foraging efficiency of cotton rats resulting in more time spent foraging. If fire ants cause cotton rats to increase their time spent foraging across an already risky landscape, cotton rats may have more encounters with other predators and experience greater predation rates. Although we did not find support for increased foraging of cotton rats influencing cotton rat populations within 45 x 45 m plots [27], it is still possible that cotton rats could respond differently to fire ants at larger spatial scales. Additionally, greater time spent foraging may increase energetic requirements and influence cotton rat stress through direct injury, fear associated with increased encounters with other predators, and/or reduced body condition [3]. These indirect effects may substantially influence an individual cotton rat’s fitness and overall health [4]. Consequently, the effects of fire ants on cotton rat foraging may be one explanation for our previous findings that fire ants have compensatory effects on cotton rat populations by reducing the survival of cotton rats in the absence of other predators [27].

Our study had the advantage of being a large-scale, replicated field experiment [45–47] that examined both the population-level and behavioral responses of the cotton rats. Our study augments and complements two prior works that used GUDs to measure the effects of fire ants on rodent foraging. Orrock and Danielson (2004) found that oldfield mice had lower GUDs at stations (spaced c. 50 m apart) when there were no signs of fire ants versus those where fire ants were present, suggesting that even at a local scale the presence and absence of fire ants pose a direct cost of foraging. In their study they took advantage of local variation in fire ant activity whereas we conducted grid-scale reductions of fire ants. Yet, despite differences in approach the results accord remarkably well.

Under a laboratory setting Holtcamp et al. (1997) measured the GUDs of deer mice in food patches with or without fire ants. With fire ants, the mice harvested seeds from trays faster, chose to handle them away from the infested patches, more strongly biased their foraging towards richer food patches, and unexpectedly left lower GUDs in the food patches. Some of these effects might not be expected to carry-over to the field where fire ants are present in the environment at large and may or may not be constantly present within the food patch itself. In our experiment, it is likely that cotton rats spent much more rather than less time in food patches on the grids with reduced fire ants, as indicated by lower GUDs and higher amounts of food harvested per individual.

It is clear that fire ants influence the foraging behaviors of cotton rats but the mechanisms underlying these impacts are still unknown. Cotton rats may use direct (e.g. being stung and/or visual) and/or indirect (e.g. olfactory) cues of fire ants to avoid risk of injury [48]. To avoid envenomation, utilizing olfactory cues to evade fire ants would be advantageous. However, the invasion of fire ants into the southeastern United States has been relatively recent [22] and cotton rats may not yet recognize the olfactory cues of fire ants. Still, native fire ants including *S*. *geminata* and *S*. *xyloni* were present on our study site prior to displacement by the red imported fire ant. If cotton rats perceive a similar risk from native fire ants then we cannot discount cotton rats generalizing olfactory recognition across these species. Nevertheless, native fire ants were never as abundant or aggressive, so it is unlikely that they would have applied the same selective pressures on cotton rats as the red imported fire ants. In a laboratory experiment, cotton rats’ use of a Y-maze was unaffected by the presence of ground fire ants [49], which supports cotton rats utilizing direct, rather than indirect, cues to avoid fire ants. Future studies should address the cues cotton rats and other rodents use to detect and avoid fire ants, determine the effects of altered foraging behaviors on risk from other predators, and if the alterations in foraging behaviors we observed lead to reduced population numbers.

## Supporting Information

S1 Table The minimum number known alive (MNKA) of hispid cotton rats (*Sigmodon hispidus*), cotton mice (*Peromyscus gossypinus*), and oldfield mice (*Peromyscus polionotus*) captured on six grids with ambient (RIFA[+]; n = 3) or reduced (RIFA[–]; n = 3) red imported fire ants (*Solenopsis invicta*) between July and September 2014. (XLSX)

S2 Table The mean amount harvested per individual (g) of millet seed harvested by hispid cotton rats (*Sigmodon hispidus*) within resource patches located on six trapping grids (n = 3 per treatment) with ambient (RIFA[+]; n = 39) or reduced (RIFA[–]; n = 39) red imported fire ant (*Solenopsis invicta*) numbers over four days from 13–15 August 2015. (XLSX)

S3 Table The giving up densities (GUDs [g]) of millet seed harvested by hispid cotton rats (*Sigmodon hispidus*) within resource patches located on six trapping grids (n = 3 per treatment) with ambient (RIFA[+]; n = 39) or reduced (RIFA[–]; n = 39) red imported fire ant (*Solenopsis invicta*) numbers over four days from 13–15 August 2015. (XLSX)

S4 Table The number of red imported fire ants (*Solenopsis invicta*) captured within resource patches located on six trapping grids (n = 3 per treatment) with ambient (RIFA[+]; n = 39) or reduced (RIFA[–]; n = 39) red imported fire ant numbers on 15 August 2015. (XLSX)

## References

[1] Preisser EL, Bolnick DI, Benard MF. Scared to death? The effects of intimidation and consumption in predator-prey interactions. Ecology. 2005;86: 501–509.

[2] Preisser EL, Bolnick DI. The Many Faces of Fear: Comparing the Pathways and Impacts of Nonconsumptive Predator Effects on Prey Populations. PLOS One. 2008;3: 1–8. 10.1371/journal.pone.0002465 PMC2409076 18560575

[3] Lima SL. Predator induced stress and behaviour. Advances in the Study of Behavior; 1998.

[4] Lima SL. Nonlethal effects in the ecology of predator-prey interactions. BioScience. 1998;48: 25–34.

[5] Lima SL, Dill LM. Behavioral decisions made under the risk of predation: a review and prospectus. Can J Zool. 1990;68: 619–640.

[6] Preisser EL, Bolnick DI, Benard MF. Scared to death? The effects of intimidation and consumption in predator-prey interactions. Ecology. 2005;86: 501–509.

[7] Brown JS, Kotler BP. Hazardous duty pay and the foraging cost of predation. Ecol Lett. 2004;7: 999–1014.

[8] Berger-Tal O, Mukherjee S, Kotler BP, Brown JS. Look before you leap: is risk of injury a foraging cost? Behav Ecol Sociobiol. 2009;63: 1821–1827. 19779627 10.1007/s00265-009-0809-3 PMC2746896

[9] Brown JS. Patch use under predation risk: I. Models and predictions. Ann Zool Fenn. 1992;29: 301–309.

[10] Schmitz OJ, Beckerman AP, O’Brien KM. Behaviorally mediated trophic cascades: Effects of predation risk on food web interactions. Ecology 1997;78: 1388–1399.

[11] Scheffers BR, Joppa LN, Pimm SL, Laurance WF. What we know and don’t know about Earth's missing biodiversity. Trends Ecol Evol. 2012;27: 501–510. doi: 10.1016/j.tree.2012.05.008 22784409 10.1016/j.tree.2012.05.008

[12] Schultz TR. In search of ant ancestors. PNAS. 2000;97: 14028–14029. 11106367 10.1073/pnas.011513798 PMC34089

[13] Holldobler B, Wilson EO. The Ants. Cambridge, MA: Harvard University Press; 1990.

[14] Lowe S, Browne M, Boudjelas S, De Poorter M. 100 of the world's worst invasive alien species: A selection from the global invasive species database. Auckland, New Zealand: The Invasive Species Specialist Group (ISSG) a specialist group of the Species Survival Commission (SSC) of the World Conservation Union (IUCN); 2000.

[15] Vanderwoude C, Lobry De Bruyn LA, House APN. Response of an open-forest ant community to invasion by the introduced ant, *Pheidole megacephala*. Austral Ecol. 2000;25: 253–259.

[16] Human KG, Gordon DM. Exploitation and interference competition between the invasive Argentine ant, *Linepithema humile*, and native ant species. Oecologia. 1996;105: 405–412. 10.1007/BF00328744 28307114

[17] Holway DA. Competitive mechanisms underlying the displacement of native ants by the invasive argentine ant. Ecology. 1999;80: 238–251.

[18] Porter SD, Savignano DA. Invasion of polygyne fire ants decimates native ants and disrupts arthropod community. Ecology. 1990;71: 2095–2106.

[19] Lubin YD. Changes in the native fauna of the Galápagos Islands following invasion by the little red fire ant, *Wasmannia auropunctata*. Biol J Linn Soc Lond. 1984;21: 229–242.

[20] Orrock JL, Danielson BJ. Rodents balancing a variety of risks: invasive fire ants and indirect and direct indicators of predation risk. Oecologia. 2004;140: 662–667. 15185138 10.1007/s00442-004-1613-4

[21] Holtcamp WN, Grant WE, Vinson SB. Patch use under predation hazard: effect of the red imported fire ant on deer mouse foraging behavior. Ecology. 1997;78: 308–317.

[22] Tschinkel WR. The fire ants. Cambridge: Belknap Press; 2006.

[23] Allen CR, Epperson DM, Garmestani AS. Red Imported fire ant impacts on wildlife: A decade of research. Am Midl Nat. 2004;152: 88–103.

[24] Lach L, Parr CL, Abott KL, editors. Ant ecology. New York: Oxford University Press; 2010.

[25] Sieg CH. Small mammals: Pests or vital components of the ecosystem. Eighth Great Plains Wildlife Damage Control Workshop Proceedings. Rapid City, SD; 1987. pp. 28–29.

[26] Ferris KP. Parental responses of hispid cotton rats (*Sigmodon hispidus*) to intrusion by red imported fire ants (*Solenopsis invicta*) into simulated nests. Thesis Texas A&M University 1994: 1–72.

[27] Long AK, Conner LM, Smith LL, McCleery RA. Effects of an invasive ant and native predators on cotton rat recruitment and survival. J Mamm. 2015;96: 1135–1141.

[28] Cameron GN, Spencer SR. Sigmodon hispidus. Mammalian Species. 2001;158: 1–9.

[29] Apperson CS, Powell EE, Browne M. Efficacy of individual mound treatments of MK-936 and Amdro against the red imported fire ant (Hymenoptera: Formicidae). J Entomol Sci. 1984;19: 508–516.

[30] Sikes RS, Gannon WL, Animal Care and Use Committee of the American Society of Mammalogists. Guidelines of the American Society of Mammalogists for the use of wild mammals in research. J Mamm. 2011;92: 235–253. 10.1093/jmammal/gyw078 PMC5909806 29692469

[31] Brown JS. Patch use as an indicator of habitat preference, predation risk, and competition. Behav Ecol Sociobiol. 1988;22: 37–47.

[32] Brown JS, Kotler BP, Mitchell WA. Foraging theory, patch use, and the structure of a Negev desert granivore community. Ecology. 1994;75: 2286–2300.

[33] R Core Team. R: A language and environment for statistical computing. URL http://www.r-project.org. Vienna, Austria; 2014.

[34] Vinson SB. Invasion of the red imported fire ant (Hymenoptera: Formicidae): spread, biology, and impact. American Entomologist. 1997;43: 23–39.

[35] Langkilde T, Freidenfelds NA. Consequences of envenomation: red imported fire ants have delayed effects on survival but not growth of native fence lizards. Wildlife Research. 2010;37: 566–573.

[36] Allen CR, Forys EA, Rice KG, Wojcik DP. Effects of fire ants (Hymenoptera: Formicidae) on hatching turtles and prevalence of fire ants on sea turtle nesting beaches in Florida. Fla Entomol. 2001;84: 250.

[37] Allen CR, Rice KG, Wojcik DP, Percival HF. Effect of red imported fire ant envenomization on neonatal American alligators. J Herpetol. 1997;31: 318–321. doi: 10.2307/1565408

[38] Allen CR, Demarais S, Lutz RS. Effects of red imported fire ants on recruitment of white-tailed deer fawns. J Wildl Manage. 1997;61: 911–916.

[39] Mueller JM, Dabbert CB, Forbes AR. Negative effects of imported fire ants on deer: the”increased movement” hypothesis. Tex J Sci. 2001;53: 87–90.

[40] Smith JE, Whelan CJ, Taylor SJ, Denight ML, Stake MM. Novel predator–prey interactions: is resistance futile? Evol Ecol Res. 2007;9: 433–446.

[41] Godbois IA, Conner LM, Warren RJ. Bobcat diet on an area managed for northern bobwhite. Proceedings of the Southeastern Association of Fish and Wildlife Agencies. 2003:57; 222–227.

[42] Collopy MW, Bildstein KL. Foraging behavior of northern harriers wintering in southeastern salt and freshwater marshes. Auk. 1987;104: 11–16.

[43] Hamilton WJ, Pollack JA. The food of some colubrid snakes from Fort Benning, Georgia. Ecology. 1956;37: 519–526.

[44] Norrdahl K, Korpimaki E. Does mobility or sex of voles affect risk of predation by mammalian predators? Ecology. 1998;79: 226–232.

[45] Macnab J. Wildlife management as scientific experimentation. Wildl Soc Bull. 1983:11; 397–401.

[46] Johnson DH. The importance of replication in wildlife research. J Wildl Manage. 2002;66: 919–932.

[47] Garton EO, Ratti JT, Giudice JH. "Research and experimental design" Techniques for wildlife investigations and management. The Wildlife Society, Bethesda, Maryland (2005): 43–71.

[48] Caro T. Antipredator defenses in birds and mammals. Chicago, IL: University of Chicago Press; 2005.

[49] Lechner KA, Ribble DO. Behavioral interactions between red imported fire ants (*Solenopsis invicta*) and three rodent species of south Texas. Southwest Nat. 1996;41: 123–128.

